# Supplementary material for: 1,25(OH)2D3 Differently Modulates the Secretory Activity of IFN-DC and IL4-DC: A Study in Cells from Healthy Donors and MS Patients
Source: Int J Mol Sci. 2023 Apr 4;24(7):6717. doi: 10.3390/ijms24076717 (PMC10094841; doi:10.3390/ijms24076717)
Supplement: Supplementary file 1 [file ijms-24-06717-s001.zip › Sanseverino, Rinaldi et al_Supplementary materials.pdf]

Supplementary materials

Supplementary Table S1

Demographic and clinical characteristics of donor groups.

	MS	MS-I	H
**No of subjects**	13	13	20
**Age (years; mean ± SD)**	37.3 ± 6	34.3 ± 10.3	41.1 ± 9.4
**Females/males (n)**	8/5	8/5	13/7
**EDSS[1] (mean ± SD)**	0.9 ± 1.5	1.1 ± 2	NA
**Disease duration (years; mean ± SD)**	6.3 ± 5.3	5.4 ± 4.5	NA

Information about age was missing for two healthy donors. NA not applicable [1] EDSS Expanded Disability Status Scale

Supplementary Table S2-legend

Anova reports of the sample sets in Figures 3-6. Sum of Squares, Degrees of Freedom, Mean Squares, F (DFn, DFd) and P values of the sources of variation are reported for each soluble mediator analyzed in culture supernatants of IFN-DC (sheet 1) and IL4-DC (sheet 2).

Supplementary Figure S1-legend

Figure S1. IL1β secretion. IL1β concentrations measured in IFN-DC (A) and IL4-DC (B) supernatants from H, MS and MS-I donors are shown as individual values. Number of subjects (n) analyzed are indicated. Black dots represent untreated cells, pink squares represent 1,25$(OH)_2$D3 treated cells.
