# Supplementary figures and images for: 1,25(OH)2D3 Differently Modulates the Secretory Activity of IFN-DC and IL4-DC: A Study in Cells from Healthy Donors and MS Patients
Source: Int J Mol Sci. 2023 Apr 4;24(7):6717. doi: 10.3390/ijms24076717 (PMC10094841; doi:10.3390/ijms24076717)

## Slide 1
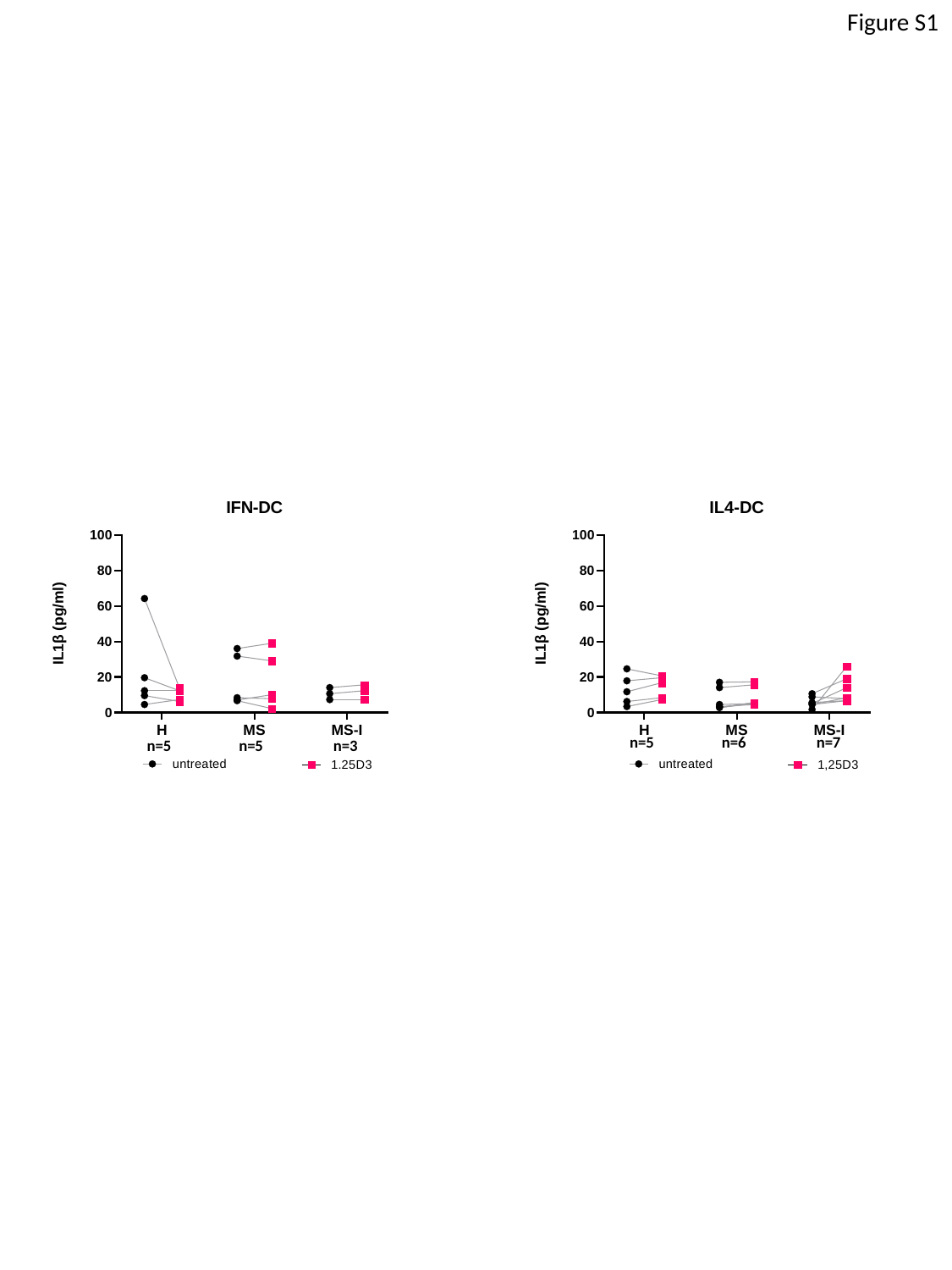

Figure S1
n=5
n=6
n=7
n=5
n=5
n=3

Supplement: Supplementary file 1 [file ijms-24-06717-s001.zip › Sanseverino, Rinaldi et al_Figure S1.pptx]
